# Genome-wide expression profiling and functional characterization of SCA28 lymphoblastoid cell lines reveal impairment in cell growth and activation of apoptotic pathways

**DOI:** 10.1186/1755-8794-6-22

**Published:** 2013-06-18

**Authors:** Cecilia Mancini, Paola Roncaglia, Alessandro Brussino, Giovanni Stevanin, Nicola Lo Buono, Helena Krmac, Francesca Maltecca, Elena Gazzano, Anna Bartoletti Stella, Maria Antonietta Calvaruso, Luisa Iommarini, Claudia Cagnoli, Sylvie Forlani, Isabelle Le Ber, Alexandra Durr, Alexis Brice, Dario Ghigo, Giorgio Casari, Anna Maria Porcelli, Ada Funaro, Giuseppe Gasparre, Stefano Gustincich, Alfredo Brusco

**Affiliations:** 1 Department of Medical Sciences, University of Torino, via Santena 19, 10126 Torino, Italy; 2 European Bioinformatics Institute, Cambridge, UK; 3 Neurobiology Sector, SISSA/ISAS, Trieste, Italy; 4 Centre de Recherche de l’Institut du Cerveau et de la Moelle épinière (INSERM / UPMC Univ. Paris 6, UMR_S975 ; CNRS 7225), Pitié-Salpêtrière Hospital, Paris, France; 5 APHP, Fédération de génétique, Pitié-Salpêtrière Hospital, Paris, France; 6 Neurogenetics team, Ecole Pratique des Hautes Etudes, Institut du Cerveau et de la Moelle épinière, CHU Pitié-Salpêtrière, Paris, France; 7 San Raffaele Scientific Institute, Vita-Salute San Raffaele University and Center for Translational Genomics and Bioinformatics, Milan-I, Italy; 8 Department of Oncology, University of Torino, Candiolo, Italy; 9 Department Medical and Surgical Sciences, Medical Genetics, University of Bologna, Bologna, Italy; 10 Department of Pharmacy and Biotechnologies (FABIT), University of Bologna, Bologna, Italy; 11 Medical Genetics Unit, “Città della Salute e della Scienza” Hospital, Torino, Italy

**Keywords:** Autosomal dominant cerebellar ataxia, Spinocerebellar ataxia, SCA28, AFG3L2, Genome-wide expression, LCLs

## Abstract

**Background:**

SCA28 is an autosomal dominant ataxia associated with *AFG3L2* gene mutations. We performed a whole genome expression profiling using lymphoblastoid cell lines (LCLs) from four SCA28 patients and six unrelated healthy controls matched for sex and age.

**Methods:**

Gene expression was evaluated with the Affymetrix GeneChip Human Genome U133A 2.0 Arrays and data were validated by real-time PCR.

**Results:**

We found 66 genes whose expression was statistically different in SCA28 LCLs, 35 of which were up-regulated and 31 down-regulated. The differentially expressed genes were clustered in five functional categories: (1) regulation of cell proliferation; (2) regulation of programmed cell death; (3) response to oxidative stress; (4) cell adhesion, and (5) chemical homeostasis. To validate these data, we performed functional experiments that proved an impaired SCA28 LCLs growth compared to controls (p < 0.005), an increased number of cells in the G0/G1 phase (p < 0.001), and an increased mortality because of apoptosis (p < 0.05). We also showed that respiratory chain activity and reactive oxygen species levels was not altered, although lipid peroxidation in SCA28 LCLs was increased in basal conditions (p < 0.05). We did not detect mitochondrial DNA large deletions. An increase of TFAM, a crucial protein for mtDNA maintenance, and of DRP1, a key regulator of mitochondrial dynamic mechanism, suggested an alteration of fission/fusion pathways.

**Conclusions:**

Whole genome expression profiling, performed on SCA28 LCLs, allowed us to identify five altered functional categories that characterize the SCA28 LCLs phenotype, the first reported in human cells to our knowledge.

## Background

Spinocerebellar ataxias or SCAs are a heterogeneous group of autosomal dominant neurodegenerative diseases with an incidence of ~ 1:30,000 and an onset which is typically in adulthood. At least 30 different subtypes and 19 causative genes have been reported [1]. For all such SCA genes, despite a well-described clinical and pathological phenotype, the molecular and cellular events that underlie neurodegeneration are still poorly understood.

SCA28 is one of the more recently identified forms, and is associated with mutations in *AFG3L2* (ATPase family gene 3-like 2) on chromosome 18p [2,3]. All mutations so far reported are missense changes, all are located in the M41-protease domain of the AFG3L2 protein with the exception of one (p.Asn432Thr) [3-5]. The disease prevalence is around 1.5% among SCA patients of European descent [2-4]. The *AFG3L2* gene encodes a subunit of the ATP-dependent metalloprotease *m*-AAA (ATPases Associated with a variety of cellular Activities), located within the inner mitochondrial membrane [6], where it exerts different functions in mitochondrial proteins processing, maturation, activation and quality control, or degradation [7-12]. The *m-*AAA displays a hexameric ring structure that in humans can be a homo-oligomer formed by AFG3L2 only, or a hetero-oligomer formed by AFG3L2 and Paraplegin (a paralog of AFG3L2 encoded by the *SPG7* gene, whose loss-of-function causes an autosomal recessive form of hereditary spastic paraplegia [12,13]). A homozygous *AFG3L2* mutation (p.Tyr616Cys) has been detected in two children affected by an early-onset severe spastic ataxia-neuropathy syndrome, characterized by severe spasticity, ataxia, and myoclonic epilepsy [14]. A recent report of *SPG7* dominant mutations in a form of optic atrophy shows that both genes may give dominant and recessive phenotypes [15].

Several groups have attempted to characterize the biochemical defects caused by alterations in the *AFG3L2* protein, albeit no study on human cells has been reported thus far. Yeast cells lacking the *m*-AAA complex transfected with mutated *AFG3L2* show a respiratory defect (i.e. incapacity to grow on a non-fermentable carbon source) related to a deficiency of respiratory chain complex IV and proteolytic impairment [3].

ATP production has been evaluated in the brain of both *Afg3l2*^*par/par*^ and *Afg3l2*^*Emv66/Emv66*^ mutant mice, which are a spontaneous mouse mutant (paralysé) carrying an Arg389Gly substitution in the conserved AAA-domain of AFG3L2 (*Afg3l2*^*par/par*^), and a null *Afg3l2* mouse model with a murine leukemia proviral insertion in exon 14 (*Afg3l2*^*Emv66/Emv66*^), respectively. In the presence of different substrates and inhibitors of the respiratory chain, results point to an assembly impairment of complexes I and III [16], while alterations in their activity and in ATP production were demonstrated in a heterozygous mouse *Afg3l2*^*+/Emv66*^, although with an onset at 4-6 months of age [17].

In this study, we characterized the genome-wide expression profile, and the cellular phenotype of human lymphoblastoid cell lines (LCLs) derived from SCA28 patients, highlighting alterations in specific pathways and functions correlated to the disease.

## Methods

### Cells lines

We obtained lymphoblastoid cell lines (LCLs) derived from SCA28 patients carrying the p.Gly671Arg (n = 3), p.Met666Arg (n = 1), p.Met666Val (n = 3), p.Met666Thr (n = 2), and p.Thr654Ile (n = 1) mutations. Clinical features of these patients have been previously described [4] and are summarized in [Additional file 1]. Fourteen control LCLs were obtained from the Human Genetics Foundation of Torino (HuGeF).

LCLs were grown at 37°C and 5% CO_2_ in RPMI-1640 medium supplemented with 10% heat-inactivated fetal bovine serum, 2 mM L-glutamine, 0.1 mg/ml streptomycin, and 100 U/ml penicillin.

### Whole genome RNA expression profiling

Total RNA was extracted from 5 × 10^6^ LCLs using the Qiaquick RNA Easy plus extraction kit (Qiagen, Mannheim, Germany) according to manufacturer's instructions. A sample of the total RNA was visualized in a 0.6% TBE 1X/agarose gel and its quality assessed using the Agilent BioAnalyser 2100 (Agilent, Palo Alto, CA). RNA was then quantified with a NanoDrop 1000 spectrophotometer (Thermo Scientific, Barrington IL, USA).

Whole-genome RNA expression study was performed on a subgroup of four SCA28 patients, each carrying a different *AFG3L2* mutation (p.Thr654Ile, p.Met666Val, p. Met666Thr, and p.Gly671Arg). Six unrelated healthy subjects matched for sex and age at sampling were used as controls.

For each sample, total RNA (6 μg) was labeled according to the standard one-cycle amplification and labeling protocol developed by Affymetrix (Santa Clara, CA, USA). Labeled cRNA was hybridized on Affymetrix GeneChip Human Genome U133A 2.0 Arrays containing probes for over 18,400 transcripts. Hybridized arrays were stained and washed on a GeneChip Fluidics Station 450 and then scanned on a GeneChip Scanner 3000 7G (Affymetrix). Cell intensity values and probe detection calls were computed from the raw array data using the Affymetrix GeneChip Operating Software (GCOS). Further data processing was performed in the R computing environment using specific packages from the BioConductor software project [18]. Robust Multi-Array Average (RMA) normalization was applied. Normalized data were filtered based on the Affymetrix detection call, so that only probes that had a Present call in at least one of the arrays were retained. Probes with low intensity values (less than 100) in all arrays were also excluded from statistical analysis.

Data were imported into the MultiExperiment Viewer (MeV) software [19], and statistical analysis was performed to detect significantly differentially expressed genes in SCA28 patients versus healthy controls, using two different tests as implemented within the MeV: Rank Product (RP) [20] and Significance Analysis of Microarrays (SAM) [21]. RP was run using 100 permutations and a False Discovery Rate (FDR) of 0.005%. The SAM test was run using 1000 permutations and a FDR of 3%. Differentially expressed genes were then specifically examined based on their Gene Ontology annotations [22] and through the use of the Database for Annotation, Visualization and Integrated Discovery (DAVID) Bioinformatics Resources [23-25].

### Validation of data by real-time RT-PCR

Genes to be validated were selected on the basis of three criteria: (i) an expression level higher than 7, (ii) fold changes higher than 1.5 or lower than 0.7 in patients *vs.* controls, and (iii) the potential interest of the gene in SCA28 pathogenesis as inferred by its function, the pathway in which it was involved and/or the functional similarity with known ataxia-related genes.

Quantitative real time PCR was used to validate array findings for 11 genes in the same LCLs batches used for microarray expression profiling (see above). The validated genes were then tested using a different RNA extraction from the same subjects (4 patients *vs*. 6 controls) to obtain a technical replicate. In addition, a third real-time PCR experiment on the same genes was performed on LCLs generated from four new different SCA28 patients obtained later [mutations p.Met666Arg, p.Met666Thr and p.Met666Val (n = 2)], as biological replicate.

Total RNA was extracted from eight SCA28 patients and eight healthy controls as described above, and 1 μg was retrotranscribed using the Transcription First Strand cDNA synthesis kit (Roche Diagnostics, Mannheim, Germany). Quantitative real-time RT-PCRs were carried out on an ABI-Prism7500 Fast instrument (Applied Biosystems) using the Taqman Gene Expression Master Mix (Applied Biosystems), Universal Probe Library (UPL) technology (Roche) and according to the manufacturer’s protocol. Primers and UPL probes used for real time PCR validation are listed in Additional file 2.

Experimental Ct values were normalized to TATA-binding protein, used as endogenous control (VIC labeled pre-designed TaqMan gene expression assays, *TBP****,*** Hs00427620_m1, Applied Biosystems). Gene expression was calculated in each sample relative to the mean of controls, using the delta-delta Ct method as described elsewhere [26]. Each sample was examined in triplicate and differences in gene expression of patients relative to controls were statistically evaluated by the Student’s *t-*test for at least three independent experiments.

### Cell growth analysis

Cell growth was determined using the MTT test [Carbonyl cyanide p‒(trifluoromethoxy) phenylhydrazone, 3‒(4,5‒dimethylthiazol) 2,5‒ diphenyl-tetrazolium bromide, Sigma-Aldrich] [27]. The growth of LCLs from six patients carrying the p.Thr654Ile (n = 1), p.Met666Thr (n = 1), p.Met666Val (n = 2), p.Met666Arg (n = 1) and p.Gly671Arg (n = 1) mutations was compared to the growth of seven LCLs from healthy controls, performing six replicates for each of the five time-points analyzed (0, 3, 6, 24, and 48 hours). After overnight incubation in RPMI with 0.5% FCS, 3 × 10^4^ cells for each time-point were washed twice in PBS solution, the pellet was resuspended in 1 ml of RPMI supplemented with 10% FCS and seeded onto 96‒well plates (150 μl/well). At each time-point, 10 μl of a 5 mg/ml MTT solution were added to each well. A negative control was included in each experiment to exclude microbial contamination.

After 1 hour incubation at 37°C in the dark the MTT solution was carefully removed, and cells were dissolved in 100 μL of DMSO for 10 minutes at 37°C. Proliferation rate of cells was determined by measuring the optical density (OD) of each sample at 570 nm with an ELISA Microplate Reader Model 680 (Biorad). To confirm growth curves with a second method, independent of mitochondrial function, we used a cytoplasmic membrane-dye (Neuro-DiO, Biotium) whose fluorescence intensity decreases as a consequence of cell divisions. The design of the experiment was the same as that of the MTT assays, and fluorescence was analyzed by a FACS Calibur flow cytometer using CellQuest software (Becton Dickinson, USA).

### Analysis of cell cycle

Cell cycle was analyzed by fluorescence labeling of DNA with Propidium Iodide (PI) [28], a stoichiometric dye, that binds in proportion to the amount of DNA present in the cell. LCLs from six healthy controls and six SCA28 patients carrying the p.Thr654Ile (n = 1), p.Met666Thr (n = 1), p.Met666Val (n = 2), p.Met666aArg (n = 1), and p.Gly671Arg (n = 1) mutations were compared at three different time-points (0, 3, and 24 h). Approximately 5 × 10^5^ cells for each time-point were subjected to density gradient centrifugation Ficoll-Paque Plus (GE Healthcare, Piscataway, NJ) to remove any dead cell and debris. Viable cells were collected at the interface and washed with PBS solution. Cells were synchronized by overnight incubation in RPMI containing 0.5% FCS, washed twice in PBS and seeded onto a 24-wells plate in 0.5 ml of RPMI 10% FCS at the final concentration of 5 × 10^5^/ well. At the designed time-points (0, 3 and 24 h), each well was washed with PBS and resuspended in 1 ml of PBS with 0.1% BSA. One ml of 70% Ethanol was added to each sample for 45 minutes at 4°C, samples were then rinsed with PBS, suspended in 0.5 ml of PBS with 0.1% BSA and 5 μl of RNaseA [10 mg/ml], and incubated 30 minutes at 37°C. One μl of PI was added to each sample for 10 minutes at 4°C in the dark, then cells populations were analyzed on a FACS Calibur flow cytometer using CellQuest software (Becton Dickinson).

### Analysis of apoptosis

Apoptosis was evaluated by fluorescence-activated cell sorting (FACS) after Annexin V/propidium iodide (PI) double staining (Biovision, San Francisco, CA, USA). LCLs from six healthy controls and six patients carrying the p.Thr654Ile (n = 1), p.Met666Arg (n = 1), p.Met666Thr (n = 1), p.Met666Val (n = 2), and p.Gly671Arg (n = 1) mutations were used [29].

Cells were washed twice with Annexin-Binding Buffer (ABB) [30], and incubated for 5 minutes in the dark with: 1) ABB alone; 2) Annexin V-FITC; 3) PI; 4) Annexin V-FITC and PI. Early apoptotic cells were only stained by Annexin V-FITC, whereas late apoptotic or necrotic cells were double-stained by Annexin V-FITC and PI. Cells were analyzed on a FACS Calibur flow cytometer using CellQuest software (Becton Dickinson). Cells in which apoptosis was induced by overnight incubation with 1 μM staurosporine were used as the positive control. To exclude that increasing apoptosis was due to starvation, the same experiments were performed without overnight incubation in RPMI with 0.5% FCS.

### ATP synthesis and respiratory chain complex evaluation

Mitochondria were isolated from three SCA28 and three controls LCLs by differential centrifugation as described [31]. Ten million cells were homogenized using a glass-Teflon homogenizer in an isotonic buffer [0.25 M sucrose, 20 mM 3-(N-morpholino) propanesulfonic acid (MOPS), pH 7.2, 1 mM EDTA] supplemented with 0.1% BSA and 0.1 mg/ml digitonin. Cell debris and nuclei were pelleted twice by centrifugation at 2,500 g for 5 minute at 4°C. Supernatants were centrifuged at 12,000 g for 25 min at 4°C and the mitochondrial pellet was resuspended in isotonic buffer (0.5 M sucrose, 20 mM MOPS, pH 7.2, 1 mM EDTA).

Respiratory chain activity was evaluated using a protocol previously described [16] and adapted to LCLs. In brief, isolated mitochondria were incubated at 37°C for 30 min in a respiratory buffer (0.25 M sucrose, 20 mM MOPS, 1 mM EDTA, 5mM inorganic phosphate, 0.1% BSA, and 1 mM ADP, pH 7.4). The function of each respiratory chain complex was tested by providing buffers containing specific substrates and inhibitors. (1) Buffer 1 (5 mM pyruvate and 1 mM malate) and (2) buffer 2 (5 mM glutamate and 1 mM malate) were used to stimulate complex I, II, III, IV, and V-dependent ATP synthesis. (3) Buffer 3 containing a complex I inhibitor (1 mM rotenone) and 10 mM succinate, was used to measure ATP production that is dependent on complex II, III, IV, and V. Finally, (4) buffer 4 [2 mM antimycin A -inhibitor of complex III- and 0.1 mM tetramethyl-p-phenylenediamine (TMPD) / 2 mM ascorbate] was used to evaluate the ATP level resulting from the activity of complex IV and V.

ATP concentration was determined with the luciferin-luciferase method as described previously [32]. In brief, isolated mitochondria were lysed with the ATP lysis buffer (200 mM NaOH and 500 nM EDTA), and an aliquot of the obtained extract was diluted with the ATP dilution buffer (100 mM NaOH and 500 nM EDTA). Twenty microliters of this mixture were added to 100 μl of the assay solution (250 mM glycylglycine, 2 mM EGTA, 2 mM MgCl_2_, 0.4 g/L BSA, 7.5 mM DTT, 15 μM luciferin, and 10 μg/ml luciferase), and the ATP content was measured using a luminometer. Three independent experiments were performed, and the significance of the means calculated using two-tailed Student’s *t*-test.

### Quantification of total cellular ATP levels

The amount of cellular ATP was measured by using the Adenosine 5'-triphosphate (ATP) Bioluminescent Assay Kit (Sigma-Aldrich, St. Louis, MO, USA), as described previously [33]. ATP content was measured in duplicate, according to manufacturer’s instructions, using an appropriate internal ATP standard for calibration. Total protein quantification was performed using classical Bradford protocol.

### Thiobarbituric acid reactive substances (TBARS) measurement

Lipid peroxidation was evaluated measuring the Thiobarbituric acid reactive substances (TBARS) levels, following published methods [34]. Cells were washed twice in PBS and the pellet resuspended in 600 μl of PBS. Five μl of Triton X-100 and 500 μl of “TBA solution” (0.375% Thiobarbituric acid and 30% Trichloroacetic Acid in 0.5 N HCl) were added to 500 μl of cells. Samples were boiled at 100°C for 20 minutes and quickly cooled by immersion in an ice bath, and centrifuged at 13000 g for 5 minutes. The absorbance of 300 μL of supernatants was read at 532 nm with a Synergy HT microplate reader (Bio-Tek Instruments, Winooski, VT), reading the absorbance at 532 nm. TBARS values were expressed as nmol/mg proteins.

### Reactive Oxygen Species evaluation

Intracellular Reactive Oxygen Species (ROS) levels were determined by using the fluorescent dye, DCFH-DA, 2,7-dichlorodihydrofluorescein diacetate (5 μM). DCFH reacts with ROS to generate a new highly fluorescent compound, dichlorofluorescein, which can be analyzed by flow cytometry. Cells were incubated with DCFH-DA (5 μM) at 37°C for 30 min, washed twice with PBS, and then measured on a FACS Calibur flow cytometer using CellQuest software (Becton Dickinson). Cells treated with H_2_O_2_ (10 μM) were used as the positive control. Three independent experiments were performed.

### Mitochondrial DNA analysis

Evaluation of mtDNA copy number was performed with an absolute quantitative TaqMan real-time PCR using the LightCycler480 (Roche), as previously detailed [35]. Long-range PCR was performed to screen for the presence of mtDNA deletions or rearrangements, as described [36].

### Western blot

Total proteins were extracted from LCLs of four patients and four healthy controls, using the RIPA Lysis Buffer [TrisHCl (50 mM), NaCl (150 mM), Triton (1%)] and quantified using standard Bradford protocol. Proteins (20-40 μg) were denaturated for 5 min at 99°C in Laemmli Buffer (Biorad) and separated by SDS–PAGE (10% or 12%) and transferred to nitrocellulose membranes (Biorad) in Tris-Glycine Buffer with 20% methanol. Western Breeze Kit (Invitrogen) was used for blocking, hybridization, secondary antibodies, and detection of antibodies, following manufacturer’s instructions. Hybridizations were performed with primary antibodies against POLγ (Abcam, ab116049, 1:1000), TFAM (Abcam, ab131607, 1:1000), VDAC (Abcam, ab15895, 1:700), DRP1 (BD Biosciences, #611112, 1:1000), OPA1 (BD Biosciences, #612606, 1:1000), MFN1 (Abcam, ab57602, 1:1000), MFN2 (Abcam, ab56889, 1:1000), ATPase-α (BD Bioscience, #612516, 1:1000) and NDUFA9 (LifeSpan Bioscience, LS-C133337 1:1000). Beta-actin antibody (Abcam Ab8227, 1:2000) or Lamin B1 antibody (Abcam, ab16048, 1:6000) were used as loading controls.

Part of the Western blot assays (DRP1, OPA1, MFN1, and MFN2) were performed after synchronization of the cells, obtained through overnight incubation in RPMI with 0.5% FCS followed by serum replacement with RPMI medium supplemented with 10% FCS for 24 hours.

## Results

### Whole genome expression analysis in SCA28 lymphoblastoid cells

To identify pathways associated with SCA28, we analyzed whole genome expression profiles in four patients’ LCLs carrying different missense mutations [p.Thr654Ile (n = 1); p.Met666Thr (n = 1); p.Met666Val (n = 1); p.Gly671Arg (n = 1)], compared to six LCLs from healthy individuals matched for sex, ethnic origin and age. We used the Affymetrix GeneChip Human Genome U133A 2.0 Arrays, which allowed to screen for 18,400 transcripts including 14,500 well-characterized genes.

**Figure 1:**
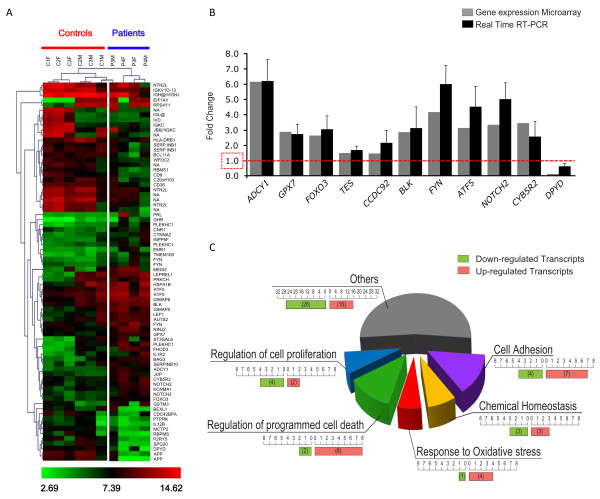
**Genome-wide expression analysis in SCA28 lymphoblasts.** (**A**) HeatMap representing 76 statistically significant probes differentially expressed in SCA28 patients (n = 4) *vs* controls (n = 6): 41 probes were up-regulated (Fold Change – FC = 2.5-10), and 35 down-regulated (FC = 0.1-0.3). Hierarchical clustering on the left shows that patients’ whole genome profiling clustered together with the exception of the patient P3F (mutation p.Gly671Arg), whose profile was slightly different. (**B**) Validation of gene expression levels by real time RT-PCR. Data obtained from microarray profiling (grey bars) were comparable to those obtained by RT-PCR on seven SCA28 lymphoblastoid cell lines (black bars with standard error). On the Y-axis, fold change as multiple of the mean value in controls (arbitrarily set to one). On the X-axis, genes tested. (**C**) Differentially expressed genes clustered in five major functional categories based on function: (1) regulation of cell proliferation; (2) regulation of programmed cell death; (3) response to oxidative stress; (4) cell adhesion, and (5) chemical homeostasis. Numbers in red and green boxes indicate the transcripts up- and down-regulated, respectively. Grey wedge indicate the remaining categories (26 genes downregulated, and 16 gene upregulated).

With Rank Product analysis we obtained a list of 76 statistically significant differentially expressed probes in patients vs. controls, 41 of which were up-regulated (Fold Change – FC = 2.5-10) and 35 down-regulated (FC = 0.1-0.3). These probes corresponded to 66 differentially expressed genes (35 up-regulated and 31 down-regulated: one gene may be tested with more than one probe) (Figure 1A) (GEO accession number GSE42406 and Additional file 3). Microarray analysis did not show any statistically significant difference in the mean expression of *AFG3L2*. The ratio between the expression of *AFG3L2* and his paralog *SPG7* was ~1.2 (9.3 vs. 7.7) both in patients and controls, in line with data previously obtained in mice nervous tissues, where *AFG3L2* is always more expressed than *SPG7*[37].

Hierarchical clustering showed that patients’ whole genome-expression profiles gathered together, with the exception of patient P3M (mutation p.Met666Val) whose profile was slightly different (Figure 1A).

We used eleven genes to validate microarray data by real-time RT-PCR (*ADCY1, GPX7, FOXO3, TES, CCDC92, BLK, FYN, ATF5, NOTCH2, CYB5R2*, and *DPYD*) in a total of eight SCA28 patients lines. Expression levels were comparable to those obtained by array profiling (Figure 1B).

The Significance Analysis of Microarrays (SAM), a more stringent statistical method, highlighted five out of the 66 genes resulting from the Rank Product test, all of which up-regulated: Adenylate cyclase 1 (brain) (*ADCY1*, FC = 6.15); Glutathione peroxidase 7 (*GPX7*, FC = 2.85); Forkhead box O3 (*FOXO3*, FC = 2.63); Testis derived transcript (3 LIM domains) (*TES*, FC = 1.48); Coiled-coil domain containing 92 (*CCDC92*, FC = 1.46).

To cluster the 66 deregulated genes for function/cellular process we integrated the Gene Ontology (GO) classification with information available through NCBI databank [38]. Five major categories were identified (Figure 1C): (1) regulation of cell proliferation; (2) regulation of programmed cell death; (3) response to oxidative stress; (4) cell adhesion, and (5) chemical homeostasis (more details in Additional file 3).

### Cell proliferation, cell cycle and cell viability assays

**Figure 2:**
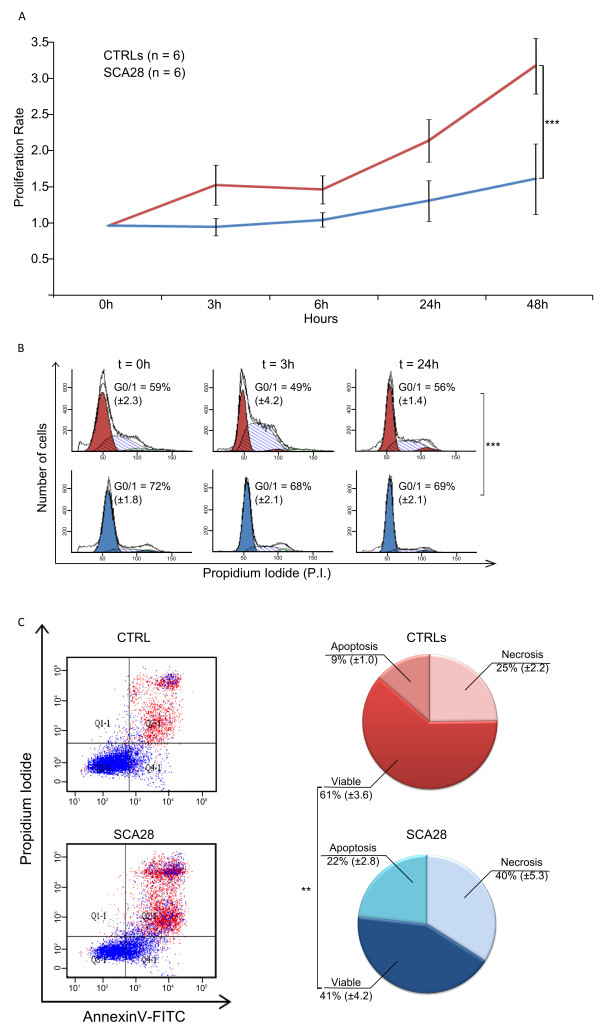
**Cell growth, cell cycle and apoptosis in SCA28 LCLs.** (**A**) Using the MTT assay, SCA28 LCLs displayed a reduced growth compared to controls (p < 0.001). (**B**) FACS analysis revealed that patients’ LCLs showed an increased number of cells in G0/G1 phase (> 15%) compared to control LCLs at any time point considered. (**C**) AnnexinV/PI FACS analysis showed that 41 ± 4.2% of SCA28 cells were viable, *vs*. 61 ± 3.6% of control cells (One-tailed Student’s *t*-test, p = 0.003).

Given the deregulation of genes involved in cell proliferation/death, we set up a series of experiments aimed at evaluating these pathways. Using the MTT and Neuro-DiO assays, SCA28 LCLs displayed a reduced growth compared to controls (p < 0.001) (Figure 2A). This observation is supported by the results of the cell cycle analysis that showed an increased percentage of cells in G0/G1 phase (> 15%) in SCA28 LCLs compared to controls. The percentage of cells in G0/G1 phase was already different at time 0 (72 ± 1.8% of SCA28 vs. 59 ± 2.3% of CTRLs, p = 0.0003) and increased at 3 hours (68 ± 2.1% vs. 49 ± 4.2%, p = 0.0006), and at 24 hours (69 ± 2.1% vs. 56 ± 1.4%, p = 0.0001) after restoration of nutrients (Figure 2B).

Cell viability measured by AnnexinV/Propidium Iodide (PI) double staining showed increased cell death in SCA28 patients with 41 ± 4.2% of viable cells *vs*. 61 ± 3.6% in controls LCLs (One-tailed Student’s *t*-test, p = 0.003; Figure 2C).

### Oxidative stress

**Figure 3:**
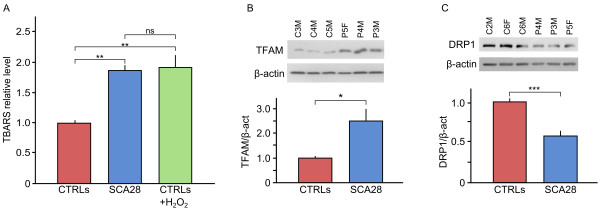
**Analysis of lipid peroxidation and mitochondrial function.** (**A**) Thiobarbituric Acid Reactive Substances (TBARS), an indirect marker of ROS was measured in SCA28 LCLs compared to controls (Kruskal-Wallis test, **: p = 0.0014; ns: not significative). SCA28 showed a two-fold increase level of TBARS suggesting the presence of hidden ROS damages, that however are not associated with an anomalous respiratory chain activity and total ATP content (not shown). (**B**) Western blot analysis of TFAM (Mitochondrial Transcription Factor A) protein showed an increase in SCA28 cell lines, suggesting an impairment in mtDNA turnover. (**C**) The analysis of selected proteins involved in mitochondrial fission/fusion showed a statistical significant reduction in DRP1 in SCA28 LCLs compared to controls LCLs (two-tailed *t* test p = 0.02).

Since oxidative stress response was one of the pathways highlighted by the gene expression analysis, we measured direct and indirect signs of ROS increase. Quantitative analysis of intracellular ROS levels with the DCFH-DA test showed that the basal levels of intracellular ROS in SCA28 patients LCLs were comparable to those of healthy controls (data not shown). We evaluated MDA levels as an indirect parameter of intracellular oxidative stress measuring the production of lipid peroxidation markers (TBARS): the results showed a ~2 fold increased level of TBARS in SCA28 LCLs *vs*. controls (Kruskal-Wallis test, p = 0.0014) (Figure 3A). The TBARS levels in SCA28 LCLs were comparable to those reached by control cells after incubation with hydrogen peroxide, a potent inducer of ROS production.

### Mitochondrial functionality

To investigate the effects of *AFG3L2* missense changes in mitochondrial metabolism, we analyzed ATP synthesis both in SCA28 cells and in isolated mitochondria in the presence of selected substrates and inhibitors. We did detect neither a significant impairment in ATP synthesis in isolated mitochondria nor a reduction in the total ATP cellular content (data not shown).

Next, we assessed the mtDNA copy number by real-time PCR to exclude mtDNA depletion. No statistically significant differences were detectable in patients compared to controls and no mtDNA deletions or large rearrangements were found (data not shown).

We measured the expression levels of six mitochondrial proteins representative of respiratory chain complexes or regulators of mtDNA replication/transcription (i.e., ATPase-α, Core2, NDUFA9, POLG1, SDHA, TFAM, VDAC). Western blot analysis of Mitochondrial Transcription Factor A (TFAM) showed a significant increase of protein levels compared to controls (Figure 3B), whereas no differences were appreciable in the other tested proteins (Additional file 4).

### Mitochondrial fission/fusion

We last explored the fission/fusion network in SCA28 LCLs, measuring DRP1, MFN1, MFN2, and OPA1 protein expression (Additional file 4). Among these, only DRP1 showed a statistically significant reduction in SCA28 LCLs compared to controls (two-tailed Student’s *t*-test, p = 0.02, Figure 3C).

## Discussion

A critical aspect of neurodegenerative disorders studies in humans is that they are hampered by the difficulty in obtaining the pathological tissues. In the attempt to overcome this limitation, other cell types such as fibroblasts, lymphocytes, or immortalized LCLs have been exploited for a wide set of experiments [39,40]. To identify disease pathways and biomarkers for fast diagnoses, microarray genome-wide expression technology, often followed by functional studies, has been used [41].

In this paper, we studied the genomic expression profile of SCA28 patients LCLs and validated the data by real-time PCR, including in a replication set of patients and controls LCLs. Differentially expressed transcripts clustered in five major functional categories: (1) regulation of cell proliferation; (2) regulation of programmed cell death; (3) response to oxidative stress; (4) cell adhesion, and (5) chemical homeostasis. Alterations of these pathways are often reported in neurodegeneration. Functional experiments confirmed an overall cellular metabolic impairment. When compared to healthy subjects, SCA28 LCLs showed an impaired growth, and an increased number of cells in the G0/G1 phase that indicated the activation of the G1 cell cycle checkpoint. Cell cycle alterations have been demonstrated in several CNS diseases including both acute damage and chronic neurodegenerative disorders [42]. Furthermore, it has been shown that the transition between G0/G1 and S phases of the cell cycle is marked by the switch from hyperfused to fragmented mitochondria [43], and mitochondrial fission is dependent on the increase of DRP1 [44]. It is therefore possible that the decreased DRP1 expression highlighted in SCA28 LCLs may affect the activity of the mitochondrial fusion/fission machinery and may be related to the cell cycle block in the G0/G1 phase.

SCA28 cells showed an increased expression of *BAG3, FOXO3* and *FYN* genes that indicates the attempt to activate an anti-apoptotic response. This, however, was not sufficient to rescue these cells, which are much more prone to undergo apoptotic death that the control cells.

On the other hand, the impairment of the oxidative stress metabolic impairment may underpin the cell cycle anomalies. Accordingly, we found increased expression of *ADCY1, FOXO3*, and *GPX7*, involved in ROS detoxification [45-47]. Increased and/or impaired ROS response is mainly the outcome of an altered mitochondrial functionality, and fits with the mitochondrial localization of AFG3L2. Moreover, it has been recently demonstrated that defective mitochondrial metabolism is detrimental for Purkinje cells, affected in spinocerebellar ataxia [39]. Intracellular ROS measured using the DCFH-DA were not increased in SCA28 cells, at least in the experimental condition adopted. However, looking at a long lasting damage induced by ROS by means of the TBARS analysis we found increased levels of lipid peroxidation. It is conceivable that the rapid metabolism of LCLs, along with the short half-life of ROS, led to a rapid turnover of oxidative species, masking the direct increase of intracellular ROS but leaving long-term by-products in the form of peroxidated lipids.

Lipid peroxidation of cell membrane phospholipids also leads to the production of several highly reactive carbonyl compounds as by-products, which may form a variety of toxic adducts including cross-linked products on amino acids or DNA bases [48]. Literature data have shown that these products are able to damage mtDNA and key mitochondrial constituents, including enzymes involved in mitochondrial respiration [49]. Nonetheless, we did not detect an altered ATP synthesis in isolated mitochondria or total cell lysates from SCA28 cells, in contrast with loss-of-function *Afg3l2*^*Emv66/Emv66*^, *Afg3l2*^*Emv66/+*^*,* and *Afg3l2*^par/par^ mouse models, where complex I and III deficits were reported [16,17]. Neither altered mtDNA copy number nor mtDNA deletions/rearrangements were furthermore detected in SCA28 LCLs, suggesting either that the level of reactive oxygen species does not trigger major mtDNA damages, or that LCLs turnover in culture does not allow for such genetic damage accumulation. In fact, it must be underlined that LCLs are not the ideal model to investigate mitochondrial functions, due to the small cytoplasmic percentage within cells. Nonetheless, these caveats reinforce the importance of our differential data between cases and controls. Interestingly, a comparable number of mtDNA molecules associated with an increase of TFAM, a crucial protein for mtDNA maintenance, may suggest an impairment in mtDNA turnover leading to the accumulation of its ‘histone-like’ protein [50,51].

**Figure 4:**
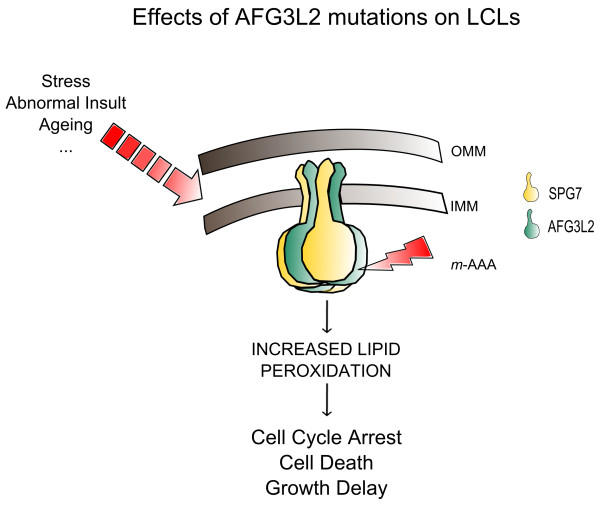
**Hypothetical model of the effect of *AFG3L2 *mutations on LCLs.** The *m*-AAA protease (here depicted as the heteromeric paraplegin/AFG3L2 isoform) is a hexameric complex within the inner mitochondrial membrane (IMM), which is involved in protein quality control. We hypothesize that mutations in *AFG3L2* result in an impairment of *m*-AAA and are connected with oxidative stress damages (lipid peroxidation), that lead to cell cycle arrest, increased cell death and consecutive growth delay. (OMM: Outer Mitochondrial Membrane)

Besides achieving the goal of characterizing SCA28 LCLs cellular hallmarks, our data allow to speculate about a possible pathogenic mechanism of SCA28. It is plausible to think that *AFG3L2* mutations likely impair the maturation of *m*-AAA substrates and/or the turnover of misfolded proteins leading to a mitochondrial damage (e.g., excess of fission and/or abnormal mtDNA turnover) and to an increased oxidative stress (increased lipid peroxidation). Extracellular stresses, such as starvation or thermal shock and ageing, may worsen this damage. The last chance for the cell is to remove damaged mitochondria “homogenizing” organelle contents through fission/fusion processes, but if this process is impaired (as it is likely in SCA28 LCLs) it drives cells towards death (Figure 4).

Other cell types may respond to an AFG3L2 altered function with a different efficiency and looking at mitochondria as an integrated subcellular system, each aspect of its function can be potentially impaired: bioenergetics, trafficking, organelle interconnectivity (fission/fusion) and protein quality control.

## Conclusions

In conclusion, whole genome expression profiling, performed on SCA28 LCLs, allowed us to identify five altered functional categories that characterize the SCA28 LCLs phenotype, the first reported in human cells to our knowledge.

The identification of specific and measurable functional aspects, such as cell cycle blockage, decreased cell growth or increased apoptosis characterizing SCA28 cells, may be used as readout for high-throughput screenings of potential drugs.

## Competing interests

The authors declare that they have no competing interests.

## Authors’ contributions

CM participated in the conception of the study and in experiments design, carried out the interpretation of expression data, performed the real-time and pathway-validation experiments, and participated in the draft of the manuscript. PR performed genome expression experiments and data analysis, and revised the manuscript. ABrussino participated in the conception of the study and in the draft of the manuscript. GS collected patients’ sample and revised the manuscript. NLB analyzed FACS data and revised the manuscript. HK performed array hybridization and revised the manuscript. FM performed ATP synthesis experiments and revised the manuscript. EG performed TBARS experiments and revised the manuscript. ABS performed mitochondrial proteins Western blotting and revised the manuscript. MAC performed total ATP content assay and revised the manuscript. LI evaluated mitochondrial DNA copy number and revised the manuscript. CC participated in in experiments design and in the draft of the manuscript. SF, IL, and AD collected patients’ sample and revised the manuscript. AD and ABrice collected patients’ clinical data and revised the manuscript. DG participated in TBARS experiment design and data analysis and revised the manuscript. GC participated in ATP synthesis experiment design and data analysis and revised the manuscript. AMP participated in total ATP content/mtDNA copy number experiments design and data analysis and revised the manuscript. AF contributed to cell cycle and cell growth experiments design and data analysis and revised the manuscript. GG contributed to mitochondrial function experiments design and data analysis, and revised the manuscript. SG contributed to genome expression experiments design and data analysis, and revised the manuscript. ABrusco participated in the conception and supervision of the study and in the draft and revision of the manuscript. All authors read and approved the final manuscript.

## Pre-publication history

The pre-publication history for this paper can be accessed here:

http://www.biomedcentral.com/1755-8794/6/22/prepub

## Supplementary Material

Additional file 1 Phenotypic features of SCA28 patients used to obtain lymphoblastoid cell lines (LCLs). Click here for file

Additional file 2 Primers and UPL probes used for real-time RT-PCR validation of microarray data. Click here for file

Additional file 3 Rank product list results and clusters for Gene Ontology categories. Click here for file

Additional file 4 **Western blot analysis of mitochondrial proteins.** ATPase-α, Core2, NDUFA9, POLG1, SDHA, VDAC (A) and MFN1, MFN2, and OPA1(B) showed no differences in patients *vs*. controls. Click here for file
