# Supplementary material for: Genome-wide expression profiling and functional characterization of SCA28 lymphoblastoid cell lines reveal impairment in cell growth and activation of apoptotic pathways
Source: BMC Med Genomics. 2013 Jun 18;6:22. doi: 10.1186/1755-8794-6-22 (PMC3689607; doi:10.1186/1755-8794-6-22)
Supplement: Additional file 1 — Phenotypic features of SCA28 patients used to obtain lymphoblastoid cell lines (LCLs). [file 1755-8794-6-22-S1.pdf]

Additional Table 1. Phenotypic features of SCA28 patients

Code	Sex	Yrs at onset	Mutation	Yrs at Exam	Severity	Gait ataxia	Limb ataxia	Dysarthria	Horiz. gaze-evoked nystagmus	Ophthalmo plegia	Lower Limbs Reflexes	Ptosis	Bilateral extensor plantar response	Other signs
**P1F**	F	28	Gly671Arg	50	Mod.	Mod.	Mod.	Mod.	No	Upward, horizontal	+	No	No	-
**P2F**	F	34	Gly671Arg	39	Mod.	Mod.	Mod.	Mod.	Yes	Horizontal	+	Yes	No	-
**P3F**	F	29	Gly671Arg	29	None	Mild	Mild	None	Yes	No	+	No	No	-
**P4F**	F	53	Met666Thr	53	Mod.	Mod.	Mod.	Severe	Yes	Vertical	N	Yes	No	Severe depression
**P5F**	F	50	Met666Val	77	Mod.	Severe	Mild	Mod.	No	Vertical, horizontal	+	Yes	Yes	-
**P1M**	M	20	Met666Thr	25	Mod.	Mod.	Mild	Mod.	Yes	No	N	No	No	Head tremor, cognitive difficulties
**P2M**	M	6	Met666Arg	10	Mild	Mild	Mild	None	No	No	N	No	No	behaviour problems
**P3M**	M	14	Met666Val	38	None	Mild	Mild	None	Yes	No	+	No	Unilateral	-
**P4M**	M	10	Thr654Ile	43	Mod.	Mod.	Mod.	Severe	Yes	No	N	No	No	Head tremor, IQ78
**P5M**	M	25	Met666Val	58	Mild	Mod.	Mod.	Mod.	Yes	Upward	+	Yes	-	-

Notes: Mod., moderate; N, normal; +, augmented;
