# Supplementary material for: Genome-wide expression profiling and functional characterization of SCA28 lymphoblastoid cell lines reveal impairment in cell growth and activation of apoptotic pathways
Source: BMC Med Genomics. 2013 Jun 18;6:22. doi: 10.1186/1755-8794-6-22 (PMC3689607; doi:10.1186/1755-8794-6-22)
Supplement: Additional file 2 — Primers and UPL probes used for real-time RT-PCR validation of microarray data. [file 1755-8794-6-22-S2.pdf]

Additional Table 2.
Primers and UPL probes (Roche Diagnostics) used for real-time RT-PCR validation of microarray data.

Gene	Forward Primer (5'->3')	Reverse Primer (5'->3')	UPL probe
ADCY1	tcctgtacctgcacatcacc	caggacagtgcgaatctgaa	47
GPX7	ccatcctgccttcaagtacc	ttccatctggggctactagg	12
FOXO3	gctaagcaggcctcatctca	gtcttgtgtcagtttgagggtct	41
TES	ttcctggaggggatagaagc	atactcagtttgcagcaatagca	3
CCDC92	cgggacttctaaaagcagtga	ttcgttctctttcactttcagttg	11
BLK	gagtggagagcctggaaatg	gagcaagaagctgcctctca	12
FYN	tgctgatctaaacgtggaaaaa	cacagcccattatctaaattcca	34
ATF5	gctcccaccttctttcttcag	caaggcgaaagtggaagact	70
NOTCH2	ggcagactggtgacttcactt	ctctcacaggtgctcccttc	8
CYB5R2	aacctgtggtacaccctgga	tcatgtcggcagtaacgaag	11
DPYD	caagagctgcaaaggaaggt	cccatcagacctgagacagtg	83
