# Supplementary figures and images for: Genome-wide expression profiling and functional characterization of SCA28 lymphoblastoid cell lines reveal impairment in cell growth and activation of apoptotic pathways
Source: BMC Med Genomics. 2013 Jun 18;6:22. doi: 10.1186/1755-8794-6-22 (PMC3689607; doi:10.1186/1755-8794-6-22)

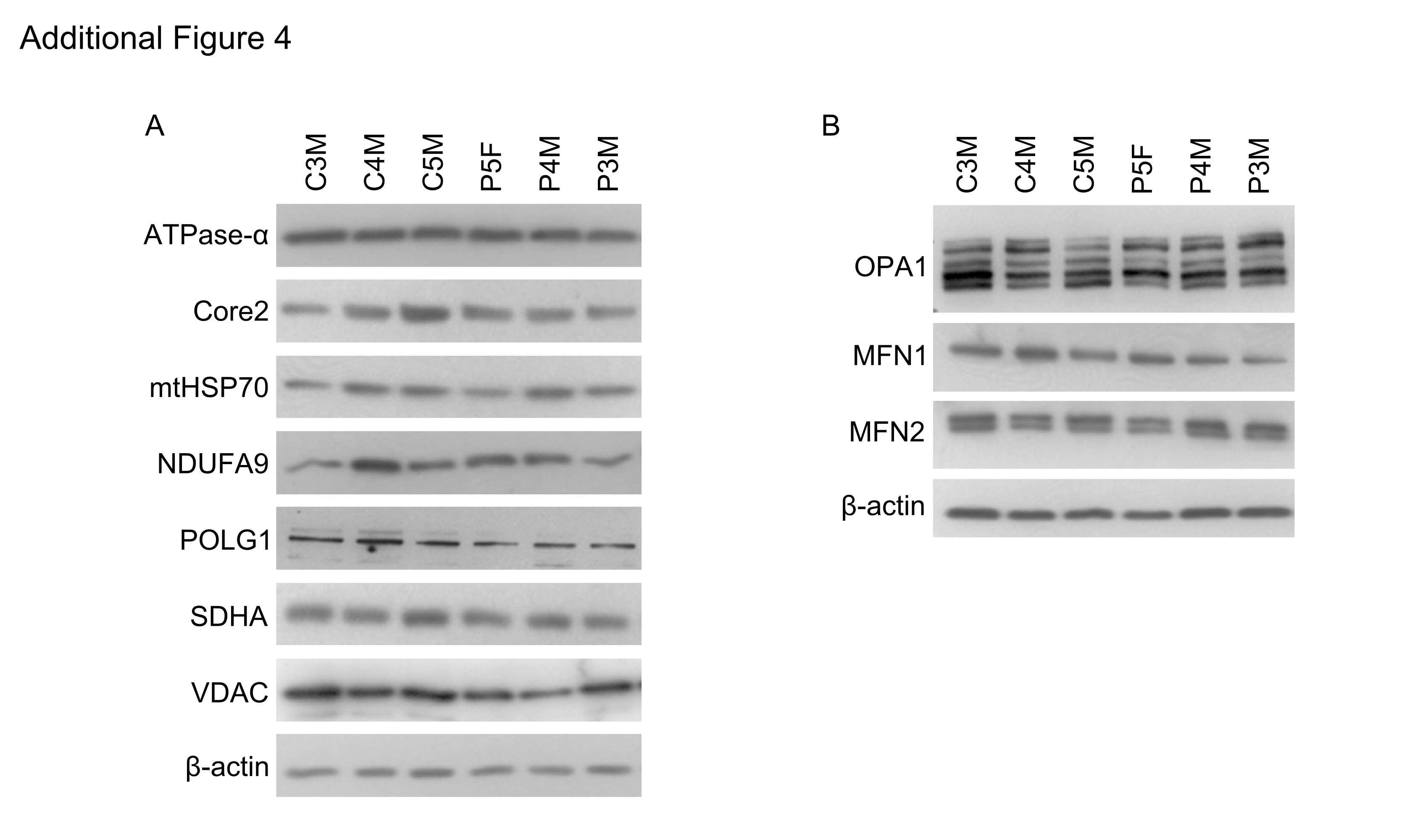

Supplement: Additional file 4 — Western blot analysis of mitochondrial proteins. ATPase-α, Core2, NDUFA9, POLG1, SDHA, VDAC (A) and MFN1, MFN2, and OPA1(B) showed no differences in patients vs. controls. [file 1755-8794-6-22-S4.png]
